# The Two-Step Strategy Could Be Inadequate and Counteracting to Diagnose Prodromal Dementia or Mild Cognitive Impairment

**DOI:** 10.3389/fnagi.2020.00229

**Published:** 2020-08-07

**Authors:** Carlo Abbate, Pietro Davide Trimarchi, Silvia Inglese, Alessia Gallucci, Emanuele Tomasini, Renzo Bagarolo, Fabrizio Giunco

**Affiliations:** ^1^IRCCS Fondazione Don Carlo Gnocchi, Milan, Italy; ^2^Geriatric Unit, Fondazione IRCCS Ca' Granda, Ospedale Maggiore Policlinico, Milan, Italy; ^3^Ph.D. Program in Neuroscience, School of Medicine and Surgery, University of Milano-Bicocca, Monza, Italy

**Keywords:** prodromal dementia, Mild Cognitive Impairment, two-step strategy, dementia diagnosis, cognitive screening test, cognitive stimulation, cognitive rehabilitation

## Introduction

The most widespread diagnostic strategy for dementia at present provides an initial brief non-specialist evaluation, usually done in a primary care setting, and, in the case of positive detection at the first visit, subsequent multidimensional diagnostic workup in a secondary care setting by a specialist. In the case of a negative outcome at the first visit, the patient is invited for a subsequent brief evaluation, usually 1 year later (Cordell et al., 2013). The same scheme of a further brief evaluation at 1-year intervals repeats whenever the brief visit results in a negative evaluation for dementia. The idea of an initial brief non-specialist evaluation has potential benefits when multiple aspects are considered, such as high cost of time-consuming specialist evaluations, the limited number of dementia specialists (Hlavka et al., 2018), high prevalence of dementia (Prince et al., 2018), and the current high number of individuals with undiagnosed dementia (Prince et al., 2018). Besides, its value has been supported by studies reporting an increased number of dementia diagnoses by adopting such a strategy (Riley McCarten et al., 2012). Accordingly, this two-step strategy is currently recommended in dementia diagnosis (Cordell et al., 2013; Pink et al., 2018).

The prodromal stage of dementia, corresponding to the Mild Cognitive Impairment (MCI) stage, has become a major focus of clinical care and research (Petersen et al., 1999; Petersen, 2004; Vos et al., 2015). In particular, MCI is a syndrome recognized at high risk for subsequent development of progressive dementia. Approximately 50–75% of the patients presenting with MCI may then develop dementia (Alcove Project, 2013). Accordingly, diagnosis at the MCI stage has multiple recognized advantages, especially when the underlying disease is identified (e.g., implementing early interventions including cognitive stimulation and rehabilitation, more accurately planning coordinated therapeutic plans, improving the management of patient symptoms and safety, reducing health care costs, and delaying institutionalization) (Alcove Project, 2013; Bianchetti et al., 2019). However, MCI is a heterogeneous syndrome with multiple possible underlying etiologies, not always converting into dementia, and sometimes even reversible (Petersen et al., 2018). Therefore, many authors underlined that the disclosure of the MCI diagnosis may also be associated with difficulties for clinicians (e.g., the classification of MCI does not allow to predict with certainty the clinical progression of the patients, it is complicated to propose concrete therapeutic solutions, or access to specific clinical trials, when the etiologic diagnosis of MCI is unclear), risks for patients (e.g., uncertainty about the diagnosis and the evolution of the symptoms could be a source of serious anxiety; it would be detrimental to wrongly upset the life of a subject presenting only benign memory problems), as well as ethical concerns (e.g., the available care options for MCI are quite limited to date, so there are ethical implications of such diagnosis to consider) (Alcove Project, 2013).

Independently from the discussion about risks and benefits associated with the MCI diagnosis, there seems to emerge agreement on the strategy to implement to detect and diagnose MCI. In particular, the two-step diagnostic strategy is now recommended also considering the diagnosis of dementia at a prodromal or MCI stage (Petersen et al., 2018). However, as clinicians particularly trained in the detection of early signs and symptoms of dementia, here we would like to underscore the notion that an initial brief evaluation, as implemented in a two-step strategy, could not only be inadequate to detect prodromal dementia but also counteracting.

## Why Initial Brief Evaluations Could be Inadequate to Detect Prodromal Dementia?

Because early signs and symptoms of cognitive decline are often subtle, heterogeneous, and masked (Abbate et al., 2019a), they are unlikely to be detected during a brief visit with a non-specialist, whose assessed in based on a cognitive screening test, few observations of the patient's behavior and a simplified interview with a patient's relative. The limitations of a brief non-specialist assessment for the detection of prodromal dementia are shown in Table 1.

**Table 1:** Characteristics of prodromal dementia and most appropriate clinical assessment.

**Characteristics of prodromal dementia**	**Description**	**Signs and symptoms of prodromal dementia**	**Why brief non-specialist evaluations cannot be effective**	**Why extended multidimensional specialist evaluations are necessary**
Heterogeneity (of features)	Many early features are not cognitive and affect different features of the patients' behavior (e.g., emotion, mood, movement, sleep, social conduct, impulse control, personality) in different kinds of dementia	e.g., apathy in AD; affective lability in VD; RBD, hypersomnia, fluctuations in attention, as well as complex hallucinations in DLB; personality changes, impulse control disorders, and social disinhibition in bvFTLD; autonomic dysfunction in MSA; falls in PSP; limb dystonia and apraxia in CBD	Cognitive screening tests evaluate general cognitive functioning only Brief, simplified interviews with informants or caregivers are usually not enough to cover the plurality of features	Multidimensional *ad-hoc* interviews with informants or caregivers are necessary to cover the plurality of features
Heterogeneity (of cognitive impairment)	There is often a focal cognitive impairment associated within a single domain. Different cognitive functions are selectively affected in different prodromal dementias	e.g., anterograde amnesia in classical AD; language impairment in PPA; semantic impairment in SD; executive impairment in VD, bvFTLD or frontal variant AD; visuospatial and/or visuoperceptive impairment both in DLB and PCA; limb apraxia in CBD	Screening tests evaluate in a preliminary way general cognitive functioning or a unique cognitive domain	Extended neuropsychological examinations by multiple tests assessing various cognitive domains in detail are necessary to cover all possible cognitive impairments found in prodromal dementias
Subtlety	Early signs are often mild and infrequent	e.g., repetitions in the discourse is a sign suggestive of impaired recent memory that sometimes emerges after 15–20 min of dialogue with the patient (Abbate et al., 2019a); sometimes a unique or a few phonemic paraphasias, that are sufficient to suspect dysphasia, emerged during a long full assessment (Abbate et al., 2019a)	Brief observation cannot be enough to detect mild and infrequent signs	Prolonged clinical observation is necessary to allow signs emerge and to note the mild and infrequent signs
Strangeness	Some signs and symptoms might be uncommon and peculiar	e.g., misidentifications and reduplications, confabulations, déjà vecù, transpositions, attentive captures, motor neglect (Abbate et al., 2019a)	Non-specialists may not be familiar with uncommon and peculiar signs and symptoms	Long training in dementia diagnosis is indispensable to be able to detect and report uncommon and peculiar signs and symptoms
Non-specificity (different diseases)	Some signs and symptoms are not specific to dementia but are common features also in different diseases, especially psychiatric diseases	e.g.: apathy in AD, affective lability in VD, depression in VD and DLB, delusions and hallucinations in DLB, obsessions, compulsions, and mania in FTLD	Non-specialists could interpret non-specific signs and symptoms as related to psychiatric diseases instead of prodromal dementia	Clinical experience is needed to correctly interpret non-specific signs and symptoms as related to prodromal dementia
Non-specificity (normal aging)	Some signs and symptoms are similar to features associated with the physiological decline in elderly	e.g., psychomotor slowness, difficulties in recent memory, reduction of spatial attention, difficulties on lexical retrieval	Non-specialists could interpret as normal some pathological signs and symptoms	Clinical experience is required to disentangle pathological signs and symptoms from physiological features by aging
Opaqueness	Some signs and symptoms do not manifest themselves in the patient's behavior	e.g., provoked confabulations, semantic deficits, simultanagnosia, stuck in set perseverations (Abbate et al., 2019a)	Non-specialists may not know how to provoke some signs and symptoms; in addition, the simple clinical observation is not enough	A specialist assessment including specific tests or *ad-hoc* interviews can provoke some signs and symptoms of prodromal dementia
Limited duration	Given dementia is characterized by progression, the prodromal stage might be short	e.g., cumulative dementia incidence was 14.9% in individuals with MCI older than age 65 years followed for 2 years. The relative risk to develop dementia if MCI is present is 3.3 at 2–5 years (Petersen et al., 2018)	Estimated cases of MCI in Europe (EU-28), considering people with age between 60 and 85 years old (i.e., 119, 327, 1,867 cases in 2018) (Statistical Office of the European Union Eurostat, 2019), and prevalence data for MCI are 13.616.081 (Petersen et al., 2018). In this context, a 10% false negative rate corresponds to ~1,361,608 people with prodromal dementia undetected at a first screening evaluation At more local level, considering the Italian population from 60 to 85 years old (15, 324, 021) (Statistical Office of the European Union Eurostat, 2019), and prevalence data for MCI (Petersen et al., 2018), a 10% false negative rate corresponds to 180,793 people with prodromal dementia undetected at the first screening	A specialist multidimensional evaluation can avoid many false negatives at the first assessment, thereby limiting the number of delayed and lost diagnoses

*MCI, mild cognitive impairment; AD, Alzheimer's disease; VD, vascular dementia; RBD, REM-behavior disorder; DLB, dementia with Lewy bodies; bvFTLD, behavioral variant of frontotemporal lobe dementia; PCA, posterior cortical atrophy; MSA, multiple system atrophy; PSP, progressive supranuclear palsy; CBD, corticobasal degeneration; PPA, primary progressive aphasia; SD, semantic dementia*.

The early detection of rare (e.g., corticobasal degeneration, CBD) or less common dementias like dementia with Lewy bodies (DLB) or frontotemporal dementia (FTD), that account for 15 and 5% of dementia cases in older people, respectively (Alzheimer's Association, 2020; Alzheimer's Research U. K. website^1^ June 2, 2020)., is expected to be difficult. Instead, the early detection of Alzheimer's disease (AD), that is the most common cause of dementia, accounting for an estimated 60–80% of cases (Alzheimer's Association, 2020; Alzheimer's Research U. K. website^1^), could appear more simple, due to the increased familiarity with its typical amnestic prodromal stage. Nonetheless, it is worth noting that prodromal AD is quite heterogeneous in clinical manifestations (Warren et al., 2012; Lam et al., 2013) and may sometimes present with different focal syndromes (e.g., posterior cortical atrophy, frontal behavioral-dysexecutive, logopenic aphasia) (Gorno-Tempini et al., 2008; Ossenkoppele et al., 2015; Crutch et al., 2017), beyond the classical and most frequent amnesic phenotype. Besides, we believed that the rate of phenotypic diversity in AD could have been underestimated currently. An increasing number of studies suggested the emergence of further phenotypes of AD well-distinct from the already known variants (e.g., right Alzheimer's disease, semantic amnesia, non-fluent aphasia) (Snowden et al., 2007; Lam et al., 2013; Abbate et al., 2019b; Woon et al., 2020). Moreover, some different diseases could show a prodromal amnestic phenotype quite similar, if not identical at first glance, to that in AD (e.g., hippocampal sclerosis HS, primary age-related tauopathy PART, limbic-predominant age-related TDP-43 encephalopathy LATE) (Crary et al., 2014; Kero et al., 2018; Nelson et al., 2019). The early detection of the subtle differences between prodromal amnestic AD and similar amnestic phenotypes of different diseases as well as the early features of the variants of AD is surely elusive at the first brief screening. But not only. Even in the case of a positive MCI detection at the first screening, we estimate that non-specialists could not feel the need of sending promptly the MCI patients to the second stage of a full assessment, so delaying the diagnostic process. We know that MCI is a syndrome with equivocal pathological significance, sometimes signaling pathological neurodegeneration, other times revealing itself a fully reversible condition.

Brief evaluations for MCI are supported overall by studies reporting that cognitive screening tests have valuable sensitivity and specificity (Tsoi et al., 2017; Breton et al., 2019; Razak et al., 2019). However, some previous studies have already shown that dementia cases discovered by screening tests included patients with dementia at intermediate and advanced stages and, more rarely, patients with prodromal dementia (Riley McCarten et al., 2012). Moreover, a cognitive screening test cannot be sufficient simply because many early signs and symptoms of prodromal dementia are not cognitive (Table 1). Furthermore, screening test sensitivity for the detection of MCI is usually presented as satisfactory; however, it rarely reaches a value larger than 90% (Tsoi et al., 2017; Breton et al., 2019; Razak et al., 2019). Accordingly, a minimum of 10% false negatives are asked to wait for another evaluation in a year. At the population level, this number of missed detections could not reveal acceptable results. For example, more than 1 million undetected prodromal dementias were asked to wait after an initial brief screening test in Europe (180,793 in Italy), considering a false negative rate of 10% (Table 1) (Statistical Office of the European Union Eurostat, 2019). Are these numbers of missed detections acceptable in a diagnostic plan provided by a national health system?

## Why Initial Brief Evaluations Could be Counteracting to Detect Prodromal Dementia?

Because patients with prodromal dementia going undetected after an initial brief visit (i.e., the false negatives) might not feel the need for a new referral for a long time, their subtle or selective disturbances are probably causing low distress. Therefore, they return to their home, probably reassured, and wait for the 12-month interval to pass, before going for the next brief visit. Considering the limited diagnostic power of the successive brief assessments, the more effective full evaluation could be further delayed by 24, 36, or even more months in some cases, making it unlikely that the patient has a high chance of receiving a prompt diagnosis of prodromal dementia. Considering the progressive course of dementia, the prodromal stage has a circumscribed duration, so there is a limited time window available to diagnose it. Repeated, ineffective brief examinations at 12-month intervals could go beyond the available time window to diagnose prodromal dementia. Therefore, a first brief non-specialist evaluation can not only delay the diagnosis of prodromal dementia but also make it likely that the opportunity is lost.

## Discussion

The two-step strategy is probably the most widespread diagnostic strategy for dementia at present. It implements the inherent way of admission of outpatients to the health care system in many countries. In particular, it is the general practitioner (GP) who usually first visits the patients with cognitive concerns, indistinctly in the suspect of dementia or MCI, in a non-specialist setting. This is a valuable aspect of a two-step strategy. It may be that many older people feel comfortable with a doctor they see and talk to regularly. Moreover, they may be more accepting of cognitive evaluation, albeit brief, by someone they know rather than a specialist they have never met before. The same may be true for family members who may act as informants. Moreover, the GP is usually aware of the general health of the patient and is likely ultimately also the person better coordinating the care for patients that are diagnosed with dementia. So, the GPs have undoubtedly a privileged position in the first detection as well as in the long-term management of dementia.

Another advantage is that diagnosing dementia in a non-specialist setting was much more inexpensive than in a specialist setting. In detail, the average cost of diagnosing a case of AD in primary care has been estimated at 753 and 849 euros, respectively, in two different studies in Sweden (Jedenius et al., 2010; Wimo et al., 2013). The corresponding cost of diagnosing dementia in specialist care was 1,298 and 1,334 euros, respectively, in the same studies. A recent study in Germany found similar results, estimating the cost of diagnosing a case of dementia in a memory clinic at 1,134 euros (Michalowsky et al., 2017). Not only, but it should also be noted that the maximal diagnostic cost of diagnosing dementia in a specialized care level, assuming all available diagnostic procedures are performed, has been estimated at over 5,000 euros (Winblad et al., 2016).

A third positive aspect to consider is that the current two-step strategy has resulted effective in diagnosing dementia (Riley McCarten et al., 2012). This positive outcome depends on different reasons. (1) Cognitive impairment and behavioral disturbances at a dementia stage are usually significant and evident so that they could be easily detected by non-specialists and reported by informants. (2) Besides, cognitive screening tests have satisfactory performance, so helping non-specialists to objectively confirm the emergence of cognitive deficits. (3) Also, the second step of the specialist full evaluation runs quite necessarily after a positive first detection, because dementia syndrome is unequivocally recognized as a pathological entity with different underlying etiologies that are to be identified by further examinations. (4) Finally, in case of a missed detection at the first screening, this error could be easily noticed and corrected. A patient with dementia returned home after resulting (false) negative at the first screening will probably search for further medical consultation after a few times, because dementia causes distress to her/him and her/his relatives. In this way, there will be soon a new opportunity available to make correct detection and diagnosis.

Despite merits reported, we believe that the two-step diagnostic strategy could present with some serious limitations when passing to consider the detection/diagnosis of prodromal dementia or MCI from the detection/diagnosis of dementia. (1) Firstly, we recognized some characteristics of prodromal dementia (Table 1) that could make the first detection of signs and symptoms more difficult than in the case of dementia, both for non-specialists as well as patient's relatives. (2) Moreover, cognitive screening tests are probably less effective in supporting the detection of MCI compared to dementia. Also, it is worth noting that many signs and symptoms of prodromal dementia are not cognitive (Table 1). (3) Besides, the second stage of the full specialist assessment could not necessarily run after a positive first detection, because MCI has a less clear pathological significance than dementia, being sometimes reversible. So, non-specialists could not feel the need to send promptly the patient to a specialist setting for further investigations. (4) Finally, in the case of a missed detection of MCI at the first step, there could not be immediate occasions to notice and timely correct the error. Cognitive impairment and behavioral disturbances at the MCI stage are, in fact, subtle or isolated, so causing less or no distress to patients and their relatives compared to dementia. Accordingly, a further visit could be postponed for a long time after a negative first detection, with the risks of not only delay the diagnosis of prodromal dementia but also make it likely that the opportunity is lost.

In light of the potential limitations reported, we are reluctant to accept the fact that patients with early suspicions of MCI are discharged after a negative first non-specialist brief evaluation without receiving the consulting by a specialist, as implemented in the current two-step strategy. Additionally, we consider it unacceptable that admission to a recommended (Hort et al., 2010) and more effective specialist multidimensional full assessment is subordinate to the outcome of the first evaluation. Instead, we believe that every patient with suspected MCI has the right to receive the consulting by a specialist at the time of the first visit, as soon as either she/he manifests subjective cognitive complaints or her/his relatives (or general practitioner) have the suspicion of cognitive impairment. Indeed, the early suspicion of cognitive impairment is very precious, so we should avoid losing this opportunity. Instead, brief preliminary evaluations with very limited diagnostic power are used and, in many cases, are unable to substantiate the suspicion. Thus, inevitably, the suspicions are silenced.

The drawbacks we found in the two-step strategy are not irrelevant, considering the very large number of people who are waiting and suffering from prodromal dementia at present (Petersen et al., 2010; Sachdev et al., 2015; Vos et al., 2015). Moreover, it is worth noting that diagnosing dementia at a prodromal or MCI stage is becoming a priority in health systems, especially after considering its economic value. Indeed, some studies have examined the potential economic benefits of early diagnosis of Alzheimer's, including from the stage of prodromal dementia or MCI, and there is general agreement that early diagnosis will save costs (Weimer and Sager, 2009; Barnett et al., 2014; Long et al., 2014; Dubois et al., 2016; Alzheimer's Association, 2018).

Coming to the possible solutions, we believed that any sustainable proposal should be aimed to improve the first detection in primary care rather than to increase expensive full assessments in specialist care (Jedenius et al., 2010; Wimo et al., 2013; Winblad et al., 2016; Michalowsky et al., 2017). At the same time, we are convinced that only dementia specialists by their advanced clinical experience in detection of signs and symptoms of prodromal dementia or MCI could adequately assist primary care doctors to achieve a full detection at the first step. So in our proposal, we envisage an intermediate stage—we may call it a “1.5 stage of full detection”—where a “frontline” dementia specialist (i.e., a behavioral neurologist, a neuropsychologist, a geriatrician, an old age psychiatrist, or an advanced practice nurse) would cooperate side by side with the primary care doctor, by revising data collected at the first screening of all cases resulted negative, and performing additional skilled clinical evaluations of the patient's cognitive, affective, and behavioral status whenever the suspect of a false negative of prodromal dementia or MCI would emerge (Abbate et al., 2016). The implementation of this new model in countries whose health care system is organized in primary and secondary care would require creating new shared spaces where close collaboration between generalists and specialists may be achieved (e.g., district memory clinics) and converting some specialists to full-time consultants for primary care services (Abbate et al., 2016). Our proposal could have acceptable cost-effectiveness, potentially reducing the number of missed first detections of prodromal dementia or MCI in virtue of the clinical consultation of a specialist, and, at the same time, saving the high cost of full assessments provided in specialist settings. In this regard, some authors have estimated that the cost of the standard procedures in diagnosing dementia in a German memory clinic, which includes clinical consultations but excludes the specific technical procedures and the other assessment used, was limited to 110 Euros (Michalowsky et al., 2017).

Our proposal fits well into the primary care-based memory clinic models that involve the intervention of specialists (Greaves and Greaves, 2011; Greaves et al., 2015) or interdisciplinary teams (Callahan et al., 2006; Lee et al., 2010, 2014, 2019; Fougère et al., 2017) working in a primary care setting to increase the ability to treat and manage dementia at the primary care level. In this context, the novelty of our proposal is that it focuses on the specific problem of the fallible detection of prodromal dementia or MCI in primary care. It is interesting to note that some of the primary care-based memory clinic projects have been ongoing for some years and have demonstrated acceptability, feasibility, and preliminary effectiveness (Callahan et al., 2006; Lee et al., 2010, 2014, 2019; Greaves and Greaves, 2011; Greaves et al., 2015; Fougère et al., 2017).

In conclusion, according to our proposal, we believe it is time to search for a solution in the education and training of many more dementia specialists, as well as developing new models of the interplay between primary and secondary care aimed to bring dementia specialists into primary care as frontline specialists (Abbate et al., 2016). Unfortunately, the current great emphasis on the role of brief cognitive screening tests by non-specialists in the primary care setting for dementia diagnosis, in our opinion, is disproportionate, exaggeratedly politically correct, and somewhat simplistic, especially considering that prodromal dementia seems to be more common, subtle, and complex.

## Author Contributions

All authors listed have made a substantial, direct and intellectual contribution to the work, and approved it for publication.

## Conflict of Interest

The authors declare that the research was conducted in the absence of any commercial or financial relationships that could be construed as a potential conflict of interest.

## Footnotes

^1^Available online at: https://www.dementiastatistics.org/statistics/different-types-of-dementia/accessed
